# Supplementary material for: A comprehensive method for the quantification of medication error probability based on fuzzy SLIM
Source: PLoS One. 2022 Feb 25;17(2):e0264303. doi: 10.1371/journal.pone.0264303 (PMC8880918; doi:10.1371/journal.pone.0264303)
Supplement: S1 File — (PDF) [file pone.0264303.s001.pdf]

Weighting of PSFs

Expert	personal factors	job-related factors	organization-related factors
E_1	(0.3,0.5,0.7)	(0.5,0.7,0.9)	(0.5,0.7,0.9)
E_2	(0.5,0.7,0.9)	(0.7,0.85,1)	(0.7,0.85,1)
E_3	(0.7,0.85,1)	(0.7,0.85,1)	(0.7,0.85,1)
E_4	(0.7,0.85,1)	(0.3,0.5,0.7)	(0.7,0.85,1)
E_5	(0.5,0.7,0.9)	(0.3,0.5,0.7)	(0.3,0.5,0.7)
E_6	(0.7,0.85,1)	(0.7,0.85,1)	(0.5,0.7,0.9)
E_7	(0.5,0.7,0.9)	(0.3,0.5,0.7)	(0.1,0.3,0.5)
E_8	(0.5,0.7,0.9)	(0.3,0.5,0.7)	(0.3,0.5,0.7)
E_9	(0.7,0.85,1)	(0.3,0.5,0.7)	(0.5,0.7,0.9)
E_{10}	(0.7,0.85,1)	(0.5,0.7,0.9)	(0.3,0.5,0.7)

Weighting of subPSFs : the pairwise comparison matrix

**E_1**						
**Personal**	Knowledge	Experience	Fatigue	Physical health	Task Time (Circadian Rhythm)	
Knowledge	(1,1,1)	(2/5,1/2,2/3)	(1,1,1)	(1,1,1)	(2,5/2,3)	
Experience	(3/2,2,5/2)	(1,1,1)	(1,3/2,2)	(1,3/2,2)	(1,3/2,2)	
Fatigue	(1,1,1)	(1/2,2/3,1)	(1,1,1)	(1,1,1)	(1,1,1)	
Physical health	(1,1,1)	(1/2,2/3,1)	(1,1,1)	(1,1,1)	(1,1,1)	
Task Time (Circadian Rhythm)	(1/3,2/5,1/2)	(1/2,2/3,1)	(1,1,1)	(1,1,1)	(1,1,1)	
**Job**	Workload	Procedures	The physical environment	Housekeeping	Transparency of responsibilities	Time available
Workload	(1,1,1)	(1/2,2/3,1)	(1/2,2/3,1)	(1,1,1)	(2/5,1/2,2/3)	(1,1,1)
Procedures	(1,3/2,2)	(1,1,1)	(1,1,1)	(1,3/2,2)	(1,1,1)	(1,3/2,2)
The physical environment	(1,3/2,2)	(1,1,1)	(1,1,1)	(1,1,1)	(1,1,1)	(1,1,1)
Housekeeping	(1,1,1)	(1/2,2/3,1)	(1,1,1)	(1,1,1)	(1/2,2/3,1)	(1,1,1)
Transparency of responsibilities	(3/2,2,5/2)	(1,1,1)	(1,1,1)	(1,3/2,2)	(1,1,1)	(1,3/2,2)

Time available	(1,1,1)	(1/2,2/3,1)	(1,1,1)	(1,1,1)	(1/2,2/3,1)	(1,1,1)
**Organization**	Patient safety climate	Safety culture	Training	Communication between staff	Supervising staff	Error Management Culture
Patient safety climate	(1,1,1)	(1,1,1)	(3/2,2,5/2)	(1,1,1)	(1,3/2,2)	(1/2,2/3,1)
Safety culture	(1,1,1)	(1,1,1)	(1,3/2,2)	(1,1,1)	(1,3/2,2)	(1/2,2/3,1)
Training	(2/5,1/2,2/3)	(1/2,2/3,1)	(1,1,1)	(1/2,2/3,1)	(1/2,2/3,1)	(2/5,1/2,2/3)
Communication between staff	(1,1,1)	(1,1,1)	(1,3/2,2)	(1,1,1)	(1,3/2,2)	(1/2,2/3,1)
Supervising staff	(1/2,2/3,1)	(1/2,2/3,1)	(1,3/2,2)	(1/2,2/3,1)	(1,1,1)	(2/5,1/2,2/3)
Error Management Culture	(1,3/2,2)	(1,3/2,2)	(3/2,2,5/2)	(1,3/2,2)	(3/2,2,5/2)	(1,1,1)

**E_2**						
**Personal**	Knowledge	Experience	Fatigue	Physical health	Task Time (Circadian Rhythm)	
Knowledge	(1,1,1)	(3/2,2,5/2)	(1,1,1)	(1,1,1)	(1,1,1)	
Experience	(2/5,1/2,2/3)	(1,1,1)	(3/2,2,5/2)	(1,1,1)	(1,1,1)	
Fatigue	(1,1,1)	(2/5,1/2,2/3)	(1,1,1)	(3/2,2,5/2)	(1,1,1)	
Physical health	(1,1,1)	(1/2,2/3,1)	(2/5,1/2,2/3)	(1,1,1)	(1,1,1)	
Task Time (Circadian Rhythm)	(1,1,1)	(1,1,1)	(1,1,1)	(1,1,1)	(1,1,1)	
**Job**	Workload	Procedures	The physical environment	Housekeeping	Transparency of responsibilities	Time available
Workload	(1,1,1)	(1,3/2,2)	(1,3/2,2)	(1,1,1)	(1,1,1)	(1,1,1)
Procedures	(1/2,2/3,1)	(1,1,1)	(1,3/2,2)	(1,3/2,2)	(1,3/2,2)	(1,3/2,2)

The physical environment	(1/2,2/3,1)	(1/2,2/3,1)	(1,1,1)	(1,3/2,2)	(1,1,1)	(1,1,1)
Housekeeping	(1,1,1)	(1/2,2/3,1)	(1/2,2/3,1)	(1,1,1)	(1,1,1)	(1,1,1)
Transparency of responsibilities	(1,1,1)	(1/2,2/3,1)	(1,1,1)	(1,1,1)	(1,1,1)	(3/2,2,5/2)
Time available	(1,1,1)	(1/2,2/3,1)	(1,1,1)	(1,1,1)	(2/5,1/2,2/3)	(1,1,1)
**Organization**	Patient safety climate	Safety culture	Training	Communication between staff	Supervising staff	Error Management Culture
Patient safety climate	(1,1,1)	(1,1,1)	(1,1,1)	(1,1,1)	(1,1,1)	(1,1,1)
Safety culture	(1,1,1)	(1,1,1)	(3/2,2,5/2)	(1,1,1)	(1,3/2,2)	(1,1,1)
Training	(1,1,1)	(2/5,1/2,2/3)	(1,1,1)	(1,1,1)	(1,1,1)	(1,3/2,2)
Communication between staff	(1,1,1)	(1,1,1)	(1,1,1)	(1,1,1)	(1,3/2,2)	(1,3/2,2)
Supervising staff	(1,1,1)	(1/2,2/3,1)	(1,1,1)	(1/2,2/3,1)	(1,1,1)	(1,1,1)
Error Management Culture	(1,1,1)	(1,1,1)	(1/2,2/3,1)	(1/2,2/3,1)	(1,1,1)	(1,1,1)

**E_3**						
**Personal**	Knowledge	Experience	Fatigue	Physical health	Task Time (Circadian Rhythm)	
Knowledge	(1,1,1)	(1,1,1)	(2/7,1/3,2/5)	(1,3/2,2)	(1/3,2/5,1/2)	
Experience	(1,1,1)	(1,1,1)	(2/7,1/3,2/5)	(1,3/2,2)	(1/2,2/3,1)	
Fatigue	(5/2,3,7/2)	(5/2,3,7/2)	(1,1,1)	(5/2,3,7/2)	(1,3/2,2)	
Physical health	(1/2,2/3,1)	(1/2,2/3,1)	(2/7,1/3,2/5)	(1,1,1)	(1/2,2/3,1)	
Task Time (Circadian Rhythm)	(2,5/2,3)	(1,3/2,2)	(1/2,2/3,1)	(1,3/2,2)	(1,1,1)	

Job	Workload	Procedures	The physical environment	Housekeeping	Transparency of responsibilities	Time available
Workload	(1,1,1)	(2,5/2,3)	(2,5/2,3)	(2,5/2,3)	(2,5/2,3)	(1,3/2,2)
Procedures	(1/3,2/5,1/2)	(1,1,1)	(1/2,2/3,1)	(1/2,2/3,1)	(1/2,2/3,1)	(1/2,2/3,1)
The physical environment	(1/3,2/5,1/2)	(1,3/2,2)	(1,1,1)	(1,3/2,2)	(1,3/2,2)	(1,1,1)
Housekeeping	(1/3,2/5,1/2)	(1,3/2,2)	(1/2,2/3,1)	(1,1,1)	(1/2,2/3,1)	(1/2,2/3,1)
Transparency of responsibilities	(1/3,2/5,1/2)	(1,3/2,2)	(1/2,2/3,1)	(1,3/2,2)	(1,1,1)	(1,1,1)
Time available	(1/2,2/3,1)	(1,3/2,2)	(1,1,1)	(1,3/2,2)	(1,1,1)	(1,1,1)
**Organization**	Patient safety climate	Safety culture	Training	Communication between staff	Supervising staff	Error Management Culture
Patient safety climate	(1,1,1)	(1,3/2,2)	(1,3/2,2)	(1/2,2/3,1)	(1,3/2,2)	(2/7,1/3,2/5)
Safety culture	(1/2,2/3,1)	(1,1,1)	(2,5/2,3)	(1/2,2/3,1)	(1,3/2,2)	(2/7,1/3,2/5)
Training	(1/2,2/3,1)	(1/3,2/5,1/2)	(1,1,1)	(1/2,2/3,1)	(1,1,1)	(2/7,1/3,2/5)
Communication between staff	(1,3/2,2)	(1,3/2,2)	(1,3/2,2)	(1,1,1)	(1,3/2,2)	(2/7,1/3,2/5)
Supervising staff	(1/2,2/3,1)	(1/2,2/3,1)	(1,1,1)	(1/2,2/3,1)	(1,1,1)	(1/3,2/5,1/2)
Error Management Culture	(5/2,3,7/2)	(5/2,3,7/2)	(5/2,3,7/2)	(5/2,3,7/2)	(2,5/2,3)	(1,1,1)

**E_4**						
**Personal**	Knowledge	Experience	Fatigue	Physical health	Task Time (Circadian Rhythm)	
Knowledge	(1,1,1)	(3/2,2,5/2)	(2/7,1/3,2/5)	(2/5,1/2,2/3)	(1/2,2/3,1)	

Experience	(2/5,1/2,2/3)	(1,1,1)	(2/7,1/3,2/5)	(2/5,1/2,2/3)	(1/2,2/3,1)	
Fatigue	(5/2,3,7/2)	(5/2,3,7/2)	(1,1,1)	(1,1,1)	(1,1,1)	
Physical health	(3/2,2,5/2)	(3/2,2,5/2)	(1,1,1)	(1,1,1)	(1,1,1)	
Task Time (Circadian Rhythm)	(1,3/2,2)	(1,3/2,2)	(1,1,1)	(1,1,1)	(1,1,1)	
**Job**	Workload	Procedures	The physical environment	Housekeeping	Transparency of responsibilities	Time available
Workload	(1,1,1)	(1,3/2,2)	(1,3/2,2)	(1,1,1)	(1,1,1)	(1,1,1)
Procedures	(1/2,2/3,1)	(1,1,1)	(2,5/2,3)	(1,1,1)	(1,1,1)	(1,1,1)
The physical environment	(1/2,2/3,1)	(1/3,2/5,1/2)	(1,1,1)	(2/5,1/2,2/3)	(1/3,2/5,1/2)	(2/5,1/2,2/3)
Housekeeping	(1,1,1)	(1,1,1)	(3/2,2,5/2)	(1,1,1)	(1,1,1)	(1,1,1)
Transparency of responsibilities	(1,1,1)	(1,1,1)	(2,5/2,3)	(1,1,1)	(1,1,1)	(1,3/2,2)
Time available	(1,1,1)	(1,1,1)	(3/2,2,5/2)	(1,1,1)	(1/2,2/3,1)	(1,1,1)
**Organization**	Patient safety climate	Safety culture	Training	Communication between staff	Supervising staff	Error Management Culture
Patient safety climate	(1,1,1)	(1/3,2/5,1/2)	(3/2,2,5/2)	(3/2,2,5/2)	(3/2,2,5/2)	(1,1,1)
Safety culture	(2,5/2,3)	(1,1,1)	(2,5/2,3)	(2,5/2,3)	(5/2,3,7/2)	(3/2,2,5/2)
Training	(2/5,1/2,2/3)	(1/3,2/5,1/2)	(1,1,1)	(3/2,2,5/2)	(3/2,2,5/2)	(1,1,1)
Communication between staff	(2/5,1/2,2/3)	(1/3,2/5,1/2)	(2/5,1/2,2/3)	(1,1,1)	(1,1,1)	(2/5,1/2,2/3)
Supervising staff	(2/5,1/2,2/3)	(2/7,1/3,2/5)	(2/5,1/2,2/3)	(1,1,1)	(1,1,1)	(2/5,1/2,2/3)
Error Management Culture	(1,1,1)	(2/5,1/2,2/3)	(1,1,1)	(3/2,2,5/2)	(3/2,2,5/2)	(1,1,1)

E$_5$						
**Personal**	Knowledge	Experience	Fatigue	Physical health	Task Time (Circadian Rhythm)	
Knowledge	(1,1,1)	(1,1,1)	(5/2,3,7/2)	(1,3/2,2)	(3/2,2,5/2)	
Experience	(1,1,1)	(1,1,1)	(5/2,3,7/2)	(3/2,2,5/2)	(2,5/2,3)	
Fatigue	(2/7,1/3,2/5)	(2/7,1/3,2/5)	(1,1,1)	(1/3,2/5,1/2)	(2,5/2,3)	
Physical health	(1/2,2/3,1)	(2/5,1/2,2/3)	(2,5/2,3)	(1,1,1)	(3/2,2,5/2)	
Task Time (Circadian Rhythm)	(2/5,1/2,2/3)	(1/3,2/5,1/2)	(1/3,2/5,1/2)	(2/5,1/2,2/3)	(1,1,1)	
**Job**	Workload	Procedures	The physical environment	Housekeeping	Transparency of responsibilities	Time available
Workload	(1,1,1)	(2,5/2,3)	(2,5/2,3)	(2,5/2,3)	(3/2,2,5/2)	(3/2,2,5/2)
Procedures	(1/3,2/5,1/2)	(1,1,1)	(1,3/2,2)	(1/2,2/3,1)	(1/2,2/3,1)	(2/5,1/2,2/3)
The physical environment	(1/3,2/5,1/2)	(1/2,2/3,1)	(1,1,1)	(2/5,1/2,2/3)	(1/2,2/3,1)	(2/5,1/2,2/3)
Housekeeping	(1/3,2/5,1/2)	(1,3/2,2)	(3/2,2,5/2)	(1,1,1)	(3/2,2,5/2)	(2/5,1/2,2/3)
Transparency of responsibilities	(2/5,1/2,2/3)	(1,3/2,2)	(1,3/2,2)	(2/5,1/2,2/3)	(1,1,1)	(2/5,1/2,2/3)
Time available	(2/5,1/2,2/3)	(3/2,2,5/2)	(3/2,2,5/2)	(3/2,2,5/2)	(3/2,2,5/2)	(1,1,1)
**Organization**	Patient safety climate	Safety culture	Training	Communication between staff	Supervising staff	Error Management Culture
Patient safety climate	(1,1,1)	(2/5,1/2,2/3)	(1/2,2/3,1)	(1,3/2,2)	(3/2,2,5/2)	(3/2,2,5/2)
Safety culture	(3/2,2,5/2)	(1,1,1)	(3/2,2,5/2)	(3/2,2,5/2)	(3/2,2,5/2)	(3/2,2,5/2)
Training	(1,3/2,2)	(2/5,1/2,2/3)	(1,1,1)	(3/2,2,5/2)	(3/2,2,5/2)	(3/2,2,5/2)
Communication between staff	(1/2,2/3,1)	(2/5,1/2,2/3)	(2/5,1/2,2/3)	(1,1,1)	(1/2,2/3,1)	(2/5,1/2,2/3)

Supervising staff	(2/5,1/2,2/3)	(2/5,1/2,2/3)	(2/5,1/2,2/3)	(1,3/2,2)	(1,1,1)	(1/2,2/3,1)
Error Management Culture	(2/5,1/2,2/3)	(2/5,1/2,2/3)	(2/5,1/2,2/3)	(3/2,2,5/2)	(1,3/2,2)	(1,1,1)

**E_6**						
**Personal**	Knowledge	Experience	Fatigue	Physical health	Task Time (Circadian Rhythm)	
Knowledge	(1,1,1)	(1/3,2/5,1/2)	(2/7,1/3,2/5)	(3/2,2,5/2)	(1/3,2/5,1/2)	
Experience	(2,5/2,3)	(1,1,1)	(1/3,2/5,1/2)	(3/2,2,5/2)	(2/5,1/2,2/3)	
Fatigue	(5/2,3,7/2)	(2,5/2,3)	(1,1,1)	(3/2,2,5/2)	(1,1,1)	
Physical health	(2/5,1/2,2/3)	(2/5,1/2,2/3)	(2/5,1/2,2/3)	(1,1,1)	(1/2,2/3,1)	
Task Time (Circadian Rhythm)	(2,5/2,3)	(3/2,2,5/2)	(1,1,1)	(1,3/2,2)	(1,1,1)	
**Job**	Workload	Procedures	The physical environment	Housekeeping	Transparency of responsibilities	Time available
Workload	(1,1,1)	(1,3/2,2)	(1,3/2,2)	(1,3/2,2)	(1,1,1)	(1/2,2/3,1)
Procedures	(1/2,2/3,1)	(1,1,1)	(1,1,1)	(1,3/2,2)	(1,3/2,2)	(2/5,1/2,2/3)
The physical environment	(1/2,2/3,1)	(1,1,1)	(1,1,1)	(1,1,1)	(2/5,1/2,2/3)	(2/5,1/2,2/3)
Housekeeping	(1/2,2/3,1)	(1/2,2/3,1)	(1,1,1)	(1,1,1)	(1,3/2,2)	(1/2,2/3,1)
Transparency of responsibilities	(1,1,1)	(1/2,2/3,1)	(3/2,2,5/2)	(1/2,2/3,1)	(1,1,1)	(1/2,2/3,1)
Time available	(1,3/2,2)	(3/2,2,5/2)	(3/2,2,5/2)	(1,3/2,2)	(1,3/2,2)	(1,1,1)
**Organization**	Patient safety climate	Safety culture	Training	Communication between staff	Supervising staff	Error Management Culture
Patient safety climate	(1,1,1)	(1/2,2/3,1)	(2/5,1/2,2/3)	(2/5,1/2,2/3)	(1/2,2/3,1)	(2/7,1/3,2/5)

Safety culture	(1,3/2,2)	(1,1,1)	(1/2,2/3,1)	(1/2,2/3,1)	(1/2,2/3,1)	(1/2,2/3,1)
Training	(3/2,2,5/2)	(1,3/2,2)	(1,1,1)	(5/2,3,7/2)	(1,1,1)	(1,1,1)
Communication between staff	(3/2,2,5/2)	(1,3/2,2)	(2/7,1/3,2/5)	(1,1,1)	(1,1,1)	(1,1,1)
Supervising staff	(1,3/2,2)	(1,3/2,2)	(1,1,1)	(1,1,1)	(1,1,1)	(1,1,1)
Error Management Culture	(5/2,3,7/2)	(1,3/2,2)	(1,1,1)	(1,1,1)	(1,1,1)	(1,1,1)

**E_7**						
**Personal**	Knowledge	Experience	Fatigue	Physical health	Task Time (Circadian Rhythm)	
Knowledge	(1,1,1)	(1/3,2/5,1/2)	(1/2,2/3,1)	(2,5/2,3)	(2/7,1/3,2/5)	
Experience	(2,5/2,3)	(1,1,1)	(1/2,2/3,1)	(1,3/2,2)	(2/7,1/3,2/5)	
Fatigue	(1,3/2,2)	(1,3/2,2)	(1,1,1)	(3/2,2,5/2)	(1,3/2,2)	
Physical health	(1/3,2/5,1/2)	(1/2,2/3,1)	(2/5,1/2,2/3)	(1,1,1)	(1/3,2/5,1/2)	
Task Time (Circadian Rhythm)	(5/2,3,7/2)	(5/2,3,7/2)	(1/2,2/3,1)	(2,5/2,3)	(1,1,1)	
**Job**	Workload	Procedures	The physical environment	Housekeeping	Transparency of responsibilities	Time available
Workload	(1,1,1)	(1,3/2,2)	(1,1,1)	(1,3/2,2)	(3/2,2,5/2)	(1,1,1)
Procedures	(1/2,2/3,1)	(1,1,1)	(1,1,1)	(1,1,1)	(1,1,1)	(2/5,1/2,2/3)
The physical environment	(1,1,1)	(1,1,1)	(1,1,1)	(1,1,1)	(1,3/2,2)	(2/5,1/2,2/3)
Housekeeping	(1/2,2/3,1)	(1,1,1)	(1,1,1)	(1,1,1)	(1,1,1)	(2/5,1/2,2/3)
Transparency of responsibilities	(2/5,1/2,2/3)	(1,1,1)	(1/2,2/3,1)	(1,1,1)	(1,1,1)	(1/3,2/5,1/2)

Time available	(1,1,1)	(3/2,2,5/2)	(3/2,2,5/2)	(3/2,2,5/2)	(2,5/2,3)	(1,1,1)
**Organization**	Patient safety climate	Safety culture	Training	Communication between staff	Supervising staff	Error Management Culture
Patient safety climate	(1,1,1)	(1,1,1)	(1/3,2/5,1/2)	(2/5,1/2,2/3)	(2/5,1/2,2/3)	(2/5,1/2,2/3)
Safety culture	(1,1,1)	(1,1,1)	(2/5,1/2,2/3)	(2/5,1/2,2/3)	(2/5,1/2,2/3)	(1,1,1)
Training	(2,5/2,3)	(3/2,2,5/2)	(1,1,1)	(3/2,2,5/2)	(1,3/2,2)	(2,5/2,3)
Communication between staff	(3/2,2,5/2)	(3/2,2,5/2)	(2/5,1/2,2/3)	(1,1,1)	(1,1,1)	(1,1,1)
Supervising staff	(3/2,2,5/2)	(3/2,2,5/2)	(1/2,2/3,1)	(1,1,1)	(1,1,1)	(1,3/2,2)
Error Management Culture	(3/2,2,5/2)	(1,1,1)	(1/3,2/5,1/2)	(1,1,1)	(1/2,2/3,1)	(1,1,1)

E_8						
**Personal**	Knowledge	Experience	Fatigue	Physical health	Task Time (Circadian Rhythm)	
Knowledge	(1,1,1)	(2,5/2,3)	(1/2,2/3,1)	(2,5/2,3)	(1,1,1)	
Experience	(1/3,2/5,1/2)	(1,1,1)	(2/5,1/2,2/3)	(3/2,2,5/2)	(3/2,2,5/2)	
Fatigue	(1,3/2,2)	(3/2,2,5/2)	(1,1,1)	(2,5/2,3)	(2,5/2,3)	
Physical health	(1/3,2/5,1/2)	(2/5,1/2,2/3)	(1/3,2/5,1/2)	(1,1,1)	(1/2,2/3,1)	
Task Time (Circadian Rhythm)	(1,1,1)	(2/5,1/2,2/3)	(1/3,2/5,1/2)	(1,3/2,2)	(1,1,1)	
**Job**	Workload	Procedures	The physical environment	Housekeeping	Transparency of responsibilities	Time available
Workload	(1,1,1)	(2,5/2,3)	(3/2,2,5/2)	(1,1,1)	(1,3/2,2)	(1/3,2/5,1/2)
Procedures	(1/3,2/5,1/2)	(1,1,1)	(3/2,2,5/2)	(1/2,2/3,1)	(3/2,2,5/2)	(1,3/2,2)

The physical environment	(2/5,1/2,2/3)	(2/5,1/2,2/3)	(1,1,1)	(2/5,1/2,2/3)	(3/2,2,5/2)	(1,1,1)
Housekeeping	(1,1,1)	(1,3/2,2)	(3/2,2,5/2)	(1,1,1)	(3/2,2,5/2)	(3/2,2,5/2)
Transparency of responsibilities	(1/2,2/3,1)	(2/5,1/2,2/3)	(2/5,1/2,2/3)	(2/5,1/2,2/3)	(1,1,1)	(1/2,2/3,1)
Time available	(2,5/2,3)	(1/2,2/3,1)	(1,1,1)	(2/5,1/2,2/3)	(1,3/2,2)	(1,1,1)
**Organization**	Patient safety climate	Safety culture	Training	Communication between staff	Supervising staff	Error Management Culture
Patient safety climate	(1,1,1)	(2,5/2,3)	(3/2,2,5/2)	(3/2,2,5/2)	(3/2,2,5/2)	(3/2,2,5/2)
Safety culture	(1/3,2/5,1/2)	(1,1,1)	(2/5,1/2,2/3)	(3/2,2,5/2)	(3/2,2,5/2)	(1,3/2,2)
Training	(2/5,1/2,2/3)	(3/2,2,5/2)	(1,1,1)	(3/2,2,5/2)	(3/2,2,5/2)	(3/2,2,5/2)
Communication between staff	(2/5,1/2,2/3)	(2/5,1/2,2/3)	(2/5,1/2,2/3)	(1,1,1)	(1/3,2/5,1/2)	(2/5,1/2,2/3)
Supervising staff	(2/5,1/2,2/3)	(2/5,1/2,2/3)	(2/5,1/2,2/3)	(2,5/2,3)	(1,1,1)	(1,3/2,2)
Error Management Culture	(2/5,1/2,2/3)	(1/2,2/3,1)	(2/5,1/2,2/3)	(3/2,2,5/2)	(1/2,2/3,1)	(1,1,1)

**E_9**						
**Personal**	Knowledge	Experience	Fatigue	Physical health	Task Time (Circadian Rhythm)	
Knowledge	(1,1,1)	(2/5,1/2,2/3)	(2/5,1/2,2/3)	(2,5/2,3)	(1,1,1)	
Experience	(3/2,2,5/2)	(1,1,1)	(2,5/2,3)	(3/2,2,5/2)	(3/2,2,5/2)	
Fatigue	(3/2,2,5/2)	(1/3,2/5,1/2)	(1,1,1)	(3/2,2,5/2)	(3/2,2,5/2)	
Physical health	(1/3,2/5,1/2)	(2/5,1/2,2/3)	(2/5,1/2,2/3)	(1,1,1)	(2/5,1/2,2/3)	

Task Time (Circadian Rhythm)	(1,1,1)	(2/5,1/2,2/3)	(2/5,1/2,2/3)	(3/2,2,5/2)	(1,1,1)	
**Job**	Workload	Procedures	The physical environment	Housekeeping	Transparency of responsibilities	Time available
Workload	(1,1,1)	(2,5/2,3)	(2,5/2,3)	(2,5/2,3)	(3/2,2,5/2)	(3/2,2,5/2)
Procedures	(1/3,2/5,1/2)	(1,1,1)	(1/2,2/3,1)	(1/2,2/3,1)	(2/5,1/2,2/3)	(1/3,2/5,1/2)
The physical environment	(1/3,2/5,1/2)	(1,3/2,2)	(1,1,1)	(2/5,1/2,2/3)	(2/5,1/2,2/3)	(1/2,2/3,1)
Housekeeping	(1/3,2/5,1/2)	(1,3/2,2)	(3/2,2,5/2)	(1,1,1)	(1/2,2/3,1)	(1/2,2/3,1)
Transparency of responsibilities	(2/5,1/2,2/3)	(3/2,2,5/2)	(3/2,2,5/2)	(1,3/2,2)	(1,1,1)	(1,1,1)
Time available	(2/5,1/2,2/3)	(2,5/2,3)	(1,3/2,2)	(1,3/2,2)	(1,1,1)	(1,1,1)
**Organization**	Patient safety climate	Safety culture	Training	Communication between staff	Supervising staff	Error Management Culture
Patient safety climate	(1,1,1)	(1,3/2,2)	(3/2,2,5/2)	(2,5/2,3)	(1,1,1)	(2,5/2,3)
Safety culture	(1/2,2/3,1)	(1,1,1)	(1/2,2/3,1)	(1,3/2,2)	(2/5,1/2,2/3)	(1,1,1)
Training	(2/5,1/2,2/3)	(1,3/2,2)	(1,1,1)	(1,3/2,2)	(3/2,2,5/2)	(1,3/2,2)
Communication between staff	(1/3,2/5,1/2)	(1/2,2/3,1)	(1/2,2/3,1)	(1,1,1)	(2/5,1/2,2/3)	(3/2,2,5/2)
Supervising staff	(1,1,1)	(3/2,2,5/2)	(2/5,1/2,2/3)	(3/2,2,5/2)	(1,1,1)	(1,1,1)
Error Management Culture	(1/3,2/5,1/2)	(1,1,1)	(1/2,2/3,1)	(2/5,1/2,2/3)	(1,1,1)	(1,1,1)

**E_{10}**						
**Personal**	Knowledge	Experience	Fatigue	Physical health	Task Time (Circadian Rhythm)	
Knowledge	(1,1,1)	(2/5,1/2,2/3)	(2/5,1/2,2/3)	(1,3/2,2)	(1,1,1)	

Experience	(3/2,2,5/2)	(1,1,1)	(1/3,2/5,1/2)	(3/2,2,5/2)	(1,3/2,2)	
Fatigue	(3/2,2,5/2)	(2,5/2,3)	(1,1,1)	(2,5/2,3)	(2,5/2,3)	
Physical health	(1/2,2/3,1)	(2/5,1/2,2/3)	(1/3,2/5,1/2)	(1,1,1)	(2,5/2,3)	
Task Time (Circadian Rhythm)	(1,1,1)	(1/2,2/3,1)	(1/3,2/5,1/2)	(1/3,2/5,1/2)	(1,1,1)	
**Job**	Workload	Procedures	The physical environment	Housekeeping	Transparency of responsibilities	Time available
Workload	(1,1,1)	(2,5/2,3)	(2,5/2,3)	(2,5/2,3)	(2,5/2,3)	(3/2,2,5/2)
Procedures	(1/3,2/5,1/2)	(1,1,1)	(3/2,2,5/2)	(3/2,2,5/2)	(3/2,2,5/2)	(1,3/2,2)
The physical environment	(1/3,2/5,1/2)	(2/5,1/2,2/3)	(1,1,1)	(1,1,1)	(1/2,2/3,1)	(1/2,2/3,1)
Housekeeping	(1/3,2/5,1/2)	(2/5,1/2,2/3)	(1,1,1)	(1,1,1)	(1,3/2,2)	(3/2,2,5/2)
Transparency of responsibilities	(1/3,2/5,1/2)	(2/5,1/2,2/3)	(1,3/2,2)	(1/2,2/3,1)	(1,1,1)	(3/2,2,5/2)
Time available	(2/5,1/2,2/3)	(1/2,2/3,1)	(1,3/2,2)	(2/5,1/2,2/3)	(2/5,1/2,2/3)	(1,1,1)
**Organization**	Patient safety climate	Safety culture	Training	Communication between staff	Supervising staff	Error Management Culture
Patient safety climate	(1,1,1)	(1,3/2,2)	(3/2,2,5/2)	(3/2,2,5/2)	(3/2,2,5/2)	(1,1,1)
Safety culture	(1/2,2/3,1)	(1,1,1)	(1/2,2/3,1)	(1,3/2,2)	(2/5,1/2,2/3)	(1,3/2,2)
Training	(2/5,1/2,2/3)	(1,3/2,2)	(1,1,1)	(3/2,2,5/2)	(3/2,2,5/2)	(3/2,2,5/2)
Communication between staff	(2/5,1/2,2/3)	(1/2,2/3,1)	(2/5,1/2,2/3)	(1,1,1)	(1/3,2/5,1/2)	(2/5,1/2,2/3)
Supervising staff	(2/5,1/2,2/3)	(3/2,2,5/2)	(2/5,1/2,2/3)	(2,5/2,3)	(1,1,1)	(2,5/2,3)
Error Management Culture	(1,1,1)	(1/2,2/3,1)	(2/5,1/2,2/3)	(3/2,2,5/2)	(1/3,2/5,1/2)	(1,1,1)

Rating of subPSFs

Personal factors					
**Task/Subtask**	**Knowledge**	**Experience**	**Fatigue**	**Physical health**	**Task Time (Circadian Rhythm)**
1.1	8	9	2	8	3
1.2	8	9	2	8	3
1.3	8	9	2	8	3
1.4	8	9	2	8	8
2.1	8	9	2	8	5
2.2	8	9	2	8	6
2.3	8	9	2	8	4
2.4	8	9	2	8	4
2.5	8	9	2	8	7
2.6	8	9	2	8	3
2.7	8	9	2	8	5
3.1	8	9	2	8	9
3.2	8	9	2	8	9
3.3	8	9	2	8	6
3.4	8	9	2	8	7
3.5	8	9	2	8	7
3.6	8	9	2	8	7
3.7	8	9	2	8	8
4.1	8	9	2	8	8
4.2	8	9	2	8	6
4.3	8	9	2	8	8
4.4	8	9	2	8	8
4.5	8	9	2	8	6
4.6	8	9	2	8	5
4.7	8	9	2	8	8
5.1	8	9	2	8	5
5.2	8	9	2	8	5
5.3	8	9	2	8	5
5.4	8	9	2	8	5
5.5	8	9	2	8	5
5.6	8	9	2	8	7

Job-related factors						
**Task/Subtask**	**Workload**	**Procedures**	**The physical environment**	**Housekeeping**	**Transparency of responsibilities**	**Time available**
1.1	2	7	4	7	7	4
1.2	2	7	4	7	7	4
1.3	2	7	4	7	7	4

1.4	2	7	4	7	7	4
2.1	5	3	4	8	7	4
2.2	2	3	4	8	7	4
2.3	2	3	4	8	7	4
2.4	2	3	4	8	7	4
2.5	2	3	4	8	7	4
2.6	2	5	4	8	7	4
2.7	2	5	4	8	7	4
3.1	5	3	6	6	7	4
3.2	5	3	6	6	7	4
3.3	2	3	6	7	7	4
3.4	2	3	6	7	7	4
3.5	2	4	6	7	7	4
3.6	2	3	6	6	7	4
3.7	2	3	6	9	7	4
4.1	2	3	6	9	8	4
4.2	2	3	6	9	8	4
4.3	2	3	6	9	8	4
4.4	2	3	6	9	8	4
4.5	2	3	6	9	8	4
4.6	2	3	6	9	8	4
4.7	2	3	6	4	8	4
5.1	2	3	5	4	8	4
5.2	2	3	5	5	8	4
5.3	2	3	5	5	8	4
5.4	2	4	5	5	8	4
5.5	2	5	5	5	8	4
5.6	2	3	5	5	8	4

Organization-related factors						
**Task/Subtask**	**Patient safety climate**	**Safety culture**	**Training**	**Communication between staff**	**Supervising staff**	**Error Management Culture**
1.1	2	5	5	8	1	1
1.2	5	5	6	6	1	1
1.3	4	5	6	5	1	1
1.4	6	5	6	7	1	1
2.1	7	7	2	4	1	1
2.2	6	5	2	5	1	1
2.3	8	6	2	6	1	1
2.4	5	4	2	4	1	1
2.5	5	5	2	4	1	1
2.6	5	4	2	5	1	1
2.7	5	6	2	7	1	1
3.1	5	6	6	8	1	1
3.2	5	6	6	8	1	1
3.3	5	3	5	8	1	1
3.4	5	5	6	8	1	1
3.5	5	5	6	5	1	1
3.6	5	6	6	8	1	1
3.7	5	5	5	8	1	1
4.1	5	4	6	8	1	1

4.2	5	6	6	5	1	1
4.3	5	7	7	8	1	1
4.4	5	6	7	8	1	1
4.5	5	4	6	4	1	1
4.6	5	7	6	8	1	1
4.7	5	7	6	8	1	1
5.1	5	5	6	7	1	1
5.2	5	4	6	6	1	1
5.3	6	4	6	5	1	1
5.4	5	7	6	8	1	1
5.5	5	6	6	8	1	1
5.6	5	3	3	8	1	1
